# Memory effects of visual and olfactory landmark information in human wayfinding

**DOI:** 10.1007/s10339-023-01169-7

**Published:** 2023-11-30

**Authors:** Mira Schwarz, Kai Hamburger

**Affiliations:** Experimental Psychology and Cognitive Science, Department of Psychology and Sport Science, Justus Liebig University, Otto-Behagel-Str. 10F, 35394 Giessen, Germany

**Keywords:** Wayfinding, Recognition, Spatial cognition, Odor memory, Olfaction, Landmark

## Abstract

Non-human animals are exceptionally good at using smell to find their way through the environment. However, the use of olfactory cues for human navigation is often underestimated. Although the sense of smell is well-known for its distinct connection to memory and emotion, memory effects in human navigation using olfactory landmarks have not been studied yet. Therefore, this article compares wayfinding and recognition performance for visual and olfactory landmarks learned by 52 participants in a virtual maze. Furthermore, it is one of the first empirical studies investigating differences in memory effects on human navigation by using two separate test situations 1 month apart. The experimental task was to find the way through a maze-like virtual environment with either olfactory or visual cues at the intersections that served as decision points. Our descriptive results show that performance was above chance level for both conditions (visual and olfactory landmarks). Wayfinding performance did not decrease 1 month later when using olfactory landmarks. In contrast, when using visual landmarks wayfinding performance decreased significantly, while visual landmarks overall lead to better recognition than olfactory landmarks at both times of testing. The results demonstrate the unique character of human odor memory and support the conclusion that olfactory cues may be used in human spatial orientation. Furthermore, the present study expands the research field of human wayfinding by providing a study that investigates memory for landmark knowledge and route decisions for the visual and olfactory modality. However, more studies are required to put this important research strand forward.

**Supplementary Information:**

The online version contains supplementary material available at 10.1007/s10339-023-01169-7.

## Introduction

In marketing, odors have been used to affect the shopping behavior of customers for many years now (Emsenhuber 2009; Fründt 2010). Meanwhile, not just supermarkets but also shopping malls or leisure activities use the human sense of smell to create an appealing atmosphere for the customer. However, until today, there is not much scientific research on multimodal connections between olfactory and other senses in human wayfinding. While we already know that many animals such as ants (Steck 2012; Steck et al. 2009), dogs, and rats (Rossier and Schenk 2003) enrich their cognitive map of the environment with olfactory landmarks to improve spatial orientation, we do not yet know to which extent people benefit from using smell and olfactory cues for navigation. Besides all the classic functions of the olfactory system, which serve mainly self-preservation, such as monitoring the safety of inhaled air (Pence et al. 2014), enabling individuals to recognize food, assess its quality (Yeomans 2006), and receive warning signals (e.g., burnt smell or poisonous elements; Scherer and Quast 2001), the research in this field proposes that the sense of smell originally evolved to support spatial orientation and navigation (Dahmani et al. 2018) and, therefore, “has a major effect on how we perceive and navigate the world” (Huber et al. 2022, p.1). Jacobs’ (2012) olfactory spatial hypothesis inspired studies of human spatial orientation using odors. New discoveries, such as spatial encoding in the mouse piriform cortex (Poo et al. 2022) and the facilitation of memory consolidation in mice and humans by nasal respiration (Arshamian et al. 2018; Tort et al. 2021), are changing many of our preconceptions about the contributions of olfaction concerning the representation of space (Jacobs 2022). Wayfinding experiments on humans meanwhile demonstrated that they are indeed capable of navigating through (virtual) environments based on olfactory cues only (Hamburger and Knauff 2019; Jacobs et al. 2015). Finally, there are striking differences between olfactory and visual memory such as an unusually resistant long-term odor memory (Herz and Engen 1996) or a strong connectivity of odors and emotions (Herz et al. 2004). Further differences are discussed below. These differences imply that the olfactory system functions separately on the neurobiological level from other sensory systems and, therefore, challenge our very definition of memory systems (Herz 2016; Herz and Engen 1996; White et al. 2020). However, these differences have not yet been studied in the context of human navigation.

Therefore, we aim to contribute in filling the gap of cognitive research in olfaction-based wayfinding by (1) examining olfactory *and* visual cues independently, (2) studying wayfinding *and* memory outcomes, and (3) using a lengthy memory retention interval (1 month).

### Present research

As already mentioned, spatial cognition researchers have a strong bias toward vision (Hamburger and Herold 2021); however, in the past years, some research on olfactory wayfinding has been conducted. Porter et al. (2007) showed that two-third of their participants were able to follow a scent path in a dog-like fashion by crawling on their knees, using solely their nose for navigation. Their performance even increased through practice. Moreover, they found that the human nostrils sample spatially distinct regions which aids scent-tracking (Porter et al. 2007). These results are supported by the findings of Wu et al. (2020) which showed that this internostril difference biases participants’ perceived direction of self-motion and, therefore, indicates “that humans have a stereo sense of smell that subconsciously guides navigation” (Wu et al. 2020, p.1). In another study by Jacobs et al. (2015), humans could return to a location based on an olfactory cue. Hamburger and Knauff (2019) demonstrated that humans performed significantly above chance by using odors for wayfinding. Even though prior visual experiences seem to be essential for building a cognitive map, findings (e.g., Hamburger and Knauff 2019; Schwarz and Hamburger 2022) suggest that wayfinding does not necessarily rely on one modality only (visual, as commonly suggested) but often uses a more or less explicit combination of modalities as cues (e.g., Hamburger and Röser 2014; for a discussion on a rather implicit usage of olfactory landmark information see 4.1 Odors as Landmarks).

### Landmarks in human wayfinding

Landmark recognition tests measure landmark knowledge, which is part of spatial knowledge (Cheng et al. 2022; Presson and Montello 1988; Siegel and White 1975; Stites et al. 2020). Landmark knowledge is as important as direction memory during landmark-based wayfinding.

Several aspects are involved in spatial learning. Siegel and White (1975) identified three components of spatial knowledge: landmark knowledge, route knowledge, and survey representations. According to Siegel and White (1975), landmarks are acquired as spatial reference points. These landmarks serve as decision points for route segments and are used to develop route knowledge. The integration of route segments produces overview maps. In addition to landmark-based wayfinding, however, a route can also be memorized sequentially based on the sequence of route segments. Thus, landmarks are only one of the many aspects in spatial learning. However, when people describe a route verbally, they orient themselves to *landmarks* (Denis 1997; Denis et al. 1999; Michon and Denis 2001). Landmarks are, therefore, a central and prominent aspect of human orientation which is the reason why this work focuses only on landmark-based wayfinding.

A landmark is defined as a salient feature in the environment that supports our understanding and memory of the structure of our environment (Montello 2017) and, therefore, serves as an orientation point. Furthermore, Yesiltepe et al. (2021) highlighted the dynamic nature of environments, potentially impacting the choice and persistence of landmarks. This is especially noteworthy in the context of landmarks that are processed in sensory modalities other than vision. For instance, olfactory landmarks may become less reliable over time due to factors like shifting winds. Research largely concurs that landmarks on route and at decision points, such as intersections, are most effective in wayfinding. Further research is needed on the cognitive saliency of landmarks in different environments, tasks, and levels of familiarity (Yesiltepe et al. 2021).

For error-free orientation, both a landmark must be recognized as a salient feature of the environment, and the path direction linked to the landmark must be remembered (Presson and Montello 1988; Siegel and White 1975; Stites et al. 2020). Here, landmark recognition tests can measure landmark knowledge (e.g., Cheng et al. 2022) and wayfinding tasks capture direction memory during landmark-based wayfinding.

Additionally, we want to highlight the aspect of landmark modality. In our study, we define landmarks as cues that provide support for understanding the structure of (virtual) environments, which are placed at decision points, and can be represented in any modality (i.e., they do not necessarily need to represent discrete “objects” as often claimed).

### Odor memory

Odor memory involves the memory for odors and memories associated with or evoked by odors (Herz and Engen 1996). As early as the beginning of the twentieth century, odor-evoked memories were described several times in the literature as particularly emotional and more intense (i.e., Bolger and Titchener 1907). Newer neuroimaging techniques have revealed direct anatomical links between the olfactory cortex and the amygdala–hippocampal complex of the limbic system. Those studies have shown that the primary olfactory cortex (POC) is continuous with the anterior portion of the amygdala and projects directly to it (Carmichael et al. 1994). Forty percent of the neurons in the rodent amygdala respond to olfactory stimulation (Cain and Bindra 1972). In many experiments, the durability of long-term odor memory was tested. Unlike memory functioning for other kinds of stimuli, long-term odor memory was unusually resistant to decay (Herz and Engen 1996) and short-term memory seems to be weak or even nonexistent since there is no evidence for primacy or recency effects for odors (Gabassi and Zanuttini 1983; Herz and Engen 1996). A possible explanation might be that odors are represented unitary in memory which limits acquisition but results in minimal loss over time due to low rates of influence, i.e., interference.

Furthermore, neuronal findings suggest that olfaction also operates independently from other sensory systems in neurobiological terms (Herz and Engen 1996). For example, olfactory information is the only sensory information which is not processed in the thalamus (which among other things is responsible for sensory integration and transfer; Farbman 1992) before being projected to the cerebral cortex (Herz and Engen 1996). Moreover, the aforementioned strong link between the amygdala and POC is unique compared to all other sensory modalities, since olfaction is the only sensory modality with direct bidirectional projections between the amygdala and primary sensory cortex (Zald and Pardo 1997). Considering this and other atypical neuronal and perceptual features of olfaction, odor memory is likely to be distinct from the memory of visual or verbal stimuli and, therefore, requires further research.

### Picture superiority versus odor superiority effect

When researching and comparing memory performance of different modalities, one quickly comes across the *picture superiority effect* (e.g., Yuille 2014). This effect originally describes the phenomenon of remembering pictures better than words (Paivio and Csapo 1973). Thus, human memory seems to be extremely sensitive to the presentation of visual information (Yuille 2014). However, little is known about possible odor superiority effects, i.e., whether odors are better remembered than words or even better than pictures.

Given the evidence of the picture superiority effect, it appears that pictures evoke deeper levels of semantic processing than words (Nelson and Brooks 1973). The *dual coding theory* explains this effect as follows: Words (read or heard) are generally encoded verbally, whereas pictures are encoded visually and verbally by labeling the picture (Paivio 1979). Thus, the likelihood of accessing the image from memory increases by being represented in two codes instead of only one (Paivio 1979). When words are used to label other sensory stimuli, the picture superiority effect should extend to other nonverbal sensory stimuli such as odors and sounds, as well. Crutcher and Beer (2011) already found an auditory superiority effect analogical to the picture superiority effect, showing that sounds were better remembered than words. A possible explanation stems from the findings of Baddeley and Hitch (1974) and Tranel et al. (2003), who showed that auditory information engages both the phonological loop as well as the visuospatial sketchpad of working memory. This means that sounds, just like pictures, are also initially processed in a different modality, namely, as (mental) images.

For odors, Lawless and Engen (1977) as well as Rabin and Cain (1984) demonstrated good retention for odors and even enhanced retention when verbal labels are assigned to odors (Lyman and McDaniel 1990; Rabin and Cain 1984). These findings suggest that even odors are processed verbally, and therefore, the dual coding theory should also apply for the olfactory system.

On the contrary, studies of Engen and Ross (1973), as well as Richardson and Zucco (1989), showed that immediate retention of odors is poor and not improved by verbal labels. Furthermore, Cain (1979) showed that odors are associated rather than identified due to the difficulty of naming the correct odor label. Considering switching costs when switching between different modalities in landmark-based wayfinding, the previous research showed no performance decline when switching from auditory to visual landmarks and vice versa (Hamburger and Röser 2011). This result is consistent with the assumption of sounds and pictures being processed in the same cognitive system. In contrast, switching between olfactory and visual stimuli incurs switching costs and decreases performance (Schwarz and Hamburger 2022). According to those findings, odors and pictures do not seem to be initially processed in the same cognitive system (Schwarz and Hamburger 2022). This argues against a dual coding of odors and consequently against better recall of odor information compared to visual information from memory.

Finally, we do not want to downplay the importance and strength of our visual system, but rather question its superiority over the olfactory system in terms of memory performance in humans based on contradictory empirical findings. Here, we do not doubt that the visual sense leads to better performance than the olfactory sense, but suggest, based on existing studies, that olfactory long-term memory is more resistant to deterioration in comparison with visual long-term memory. Thus, the better memory performance for odors could compensate or even outperform the initial better performance for pictures over long periods of time.

Our experimental design should provide insights into the question whether there is a superiority of the visual system over the olfactory system in human wayfinding and whether, after a longer period of time, this initial superiority of the visual system could be compensated for by the superiority of the olfactory memory.

### Question and hypotheses

So far, only few studies have integrated odor landmark information in experiments for the purpose of providing information about their use in human olfactory wayfinding and recognition. Moreover, there has been no study known to the authors that combined this field of research with odor memory. Therefore, we want to investigate the effects of odor memory on human navigation in an experiment using either visual or olfactory landmarks in a virtual environment wayfinding and recognition task. To explore memory effects, the participants completed the experimental tasks twice at a 1-month interval.

#### Wayfinding task

There is a body of literature on the role of landmark modality in human wayfinding available from our research group (e.g., Arena and Hamburger 2022; Hamburger and Knauff 2019; Hamburger and Röser 2014; Karimpur and Hamburger 2016). Here, wayfinding performance was similar for all tested modalities (visual, acoustic, visual verbal, and olfactory). Therefore, we hypothesize that (1) wayfinding performance does not vary between the different landmark modalities (olfactory vs. visual) right after the learning phase (t1).

However, since some studies found that odor memory is more resistant to decay than other modalities, we expect that (2) wayfinding performance remains the same 1 month after learning (t2) when using olfactory landmarks. In contrast, we assume that (3) wayfinding performance in the visual condition will decrease 1 month after learning.

#### Recognition task

Given the equivocal findings suggesting a direct connection between olfaction and memory on the one hand (see *1.3 Odor Memory*) and the mixed evidence both against and in favor of dual encoding of olfactory stimuli on the other hand (see *1.4 Picture Superiority versus Odor Superiority Effect*), no clear assumptions can be derived with regard to which modality (olfactory or visual) leads to better recognition right after the learning phase (t1) and 1 month later (t2). Therefore, we here investigate (4) the difference in recognition performance between olfactory and visual landmark information.

## Materials and methods

### Participants

An a priori sample size estimation revealed that with an effect size of *f* = 0.5 and a power of 0.9, a total of 54 participants would be required to obtain a significant result with a 2-measure repeated-measures ANOVA (*α* = 0.05). In this study, we were able to test a total of 52 (37 females and 14 males, one did not indicate gender) participants at both times of testing. Nineteen of the 52 subjects were subsequently tested during the revision phase. Considering an alpha level of 0.05, the available sample size of 52 participants at two times of testing, and a desired level of power of 0.95, we identified the minimum effect size of 0.388 of an interaction effect that can be reliably detected. This means that our analysis will be able to reliably identify medium to large effects according to Cohen (2013).

Age was controlled for across groups, and the age range of the participants was 19–62 years (*M* = 25.47, SD = 9.68). Age did not significantly impact the dependent variables in the current study. Exclusion criteria included any type of restriction in the ability to smell or see and people suffering from epilepsy. Participation was voluntary, and participants either received course credits for participation if required or were able to take part in a raffle in which ten 20 € Amazon vouchers were drawn.

All participants provided informed written consent approved by a local ethics committee. The use of the landmark material and the procedure of the experiment were in accordance with the latest version (October 2013) of the Declaration of Helsinki.

### Materials

**Fig. 1:**
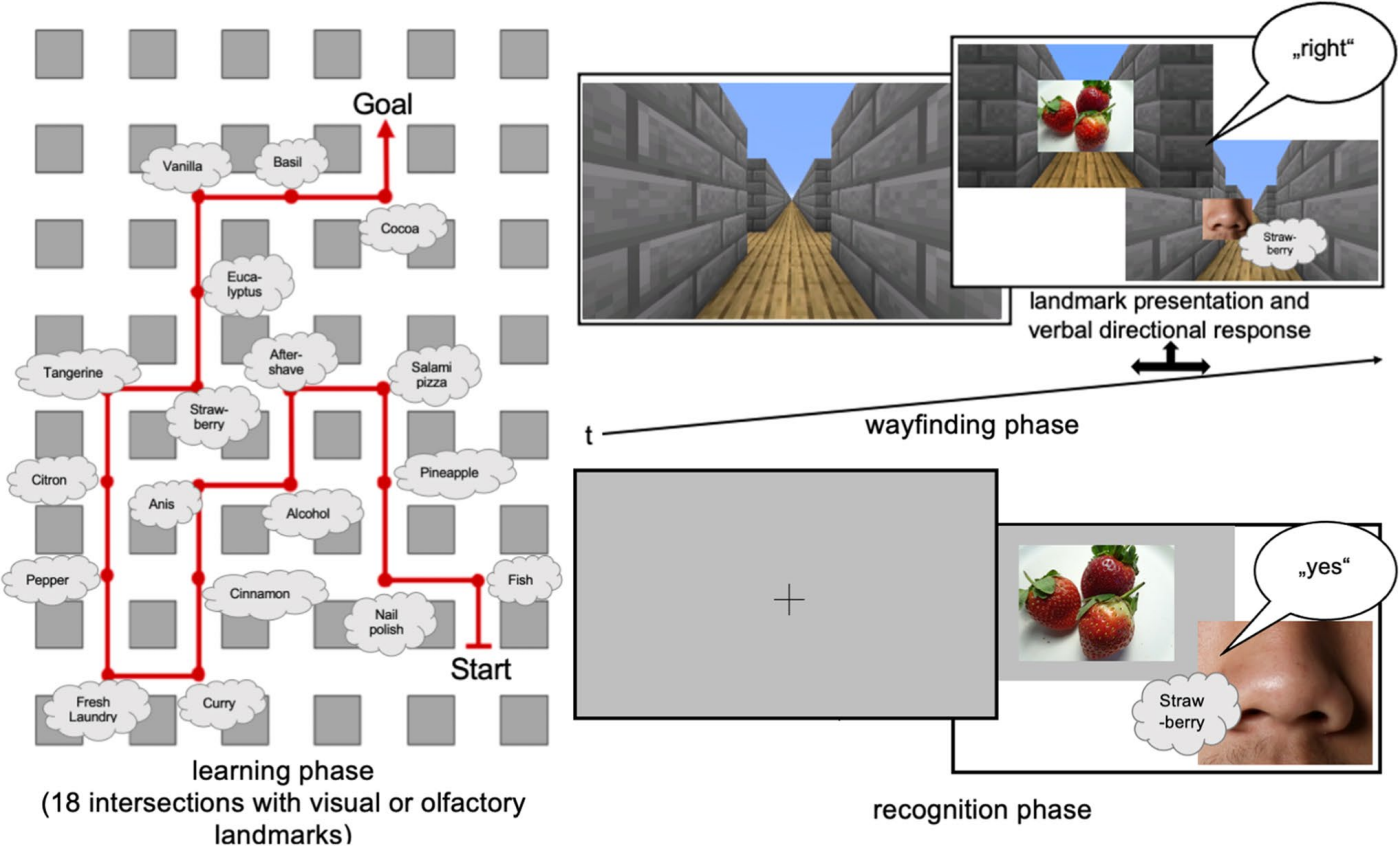
(left) Exemplary route for the *learning phase* in a virtual environment built with Minecraft ® with either olfactory or visual cues at each intersection (cloud symbol); (top right) in the *wayfinding phase* participants again saw the video sequence which was stopped at every intersection, and they had to decide—based on the specific landmark (olfactory or visual) at the intersection—whether to turn right, left, or move straight; and (bottom right) in the *recognition phase,* the 18 landmarks plus 18 distractors (olfactory or visual) were presented in random order, participants had to decide whether they already smelled/saw the cues in the learning and wayfinding phase (“yes”/”no”). Microsoft ® Word for Mac was used to create the figure

The main experiment consisted of three parts, a *learning,* a *wayfinding,* and a *recognition phase* (Fig. 1). During the learning phase, 18 odors or pictures (= *landmarks*) were presented at 18 different intersections of a virtual environment built with Minecraft ® (Mojang Synergies 2022). The virtual environment consisted of a self-built 3D maze. Two routes were presented via a head-mounted display (HTC Vive). The second route was a mirrored version of the first route and was presented to every second participant. The routes were identical for all three between-subject conditions with 12 direction changes (six right and six left) and six times straight at an intersection. Since participants could choose among three directions, the probability of choosing the correct way by chance was 1/3.

Olfactory salience (valence, arousal, and dominance) of 55 odors was already estimated in the past by Hamburger and Herold (2021) for systematic future research. For our experiment, we picked 36 of those well-documented odors as landmarks. In order to have a diverse selection of landmarks, odors with high, middle, and low valence, arousal, and dominance were considered. For the recognition test, in which 18 landmarks of the learning phase (either odors, pictures, or both) plus 18 distractor stimuli were presented in random order, stimulus pairs of equal valence, arousal, and dominance scores (according to Hamburger and Herold 2021) were generated. One of the two odors (and the matching visual stimuli) were assigned to the maze (landmark) while the other one became a distractor in the recognition task. Therefore, half of the selected odors and matching visual stimuli (*n* = 18) served as distractors, and the other half were used as visual and olfactory landmarks. The odors were stored in brown glass vials.

The matching visual landmarks were taken from private sources and the license-free stock images provider pexels.com (see Online Resource 1).

### Procedure

Fifty-two participants were pseudo-randomly assigned to each of the two conditions (olfactory unimodal: *n* = 25 and visual unimodal: *n* = 27). Both conditions had two alternating routes with three alternating stimuli sequences which allowed us to control for systematic position effects (see Online Resource 2). Participants wore the head-mounted display (HTC Vive) throughout the whole experiment.

During the *learning phase*, they watched a video sequence of the virtual maze showing a route from the first-person perspective (i.e., egocentric) including 18 landmarks, either visual or olfactory. Presentation times of all landmarks were always held constant throughout the experiment (visual landmarks were presented for 3 s; olfactory landmarks were presented for 5 s due to longer processing times). The visual landmarks were presented as pictures at each intersection (see Fig. 1); the olfactory landmarks were presented by the experimenter per hand in open vials.

After the learning phase, in the *wayfinding task*, the participants were asked to watch the video sequence from the learning phase again, but this time, the sequence stopped at each intersection. The landmarks of the wayfinding phase were presented in the identical modality and with the same presentation duration as in learning phase [e.g., olfactory landmarks in the learning phase led to olfactory stimuli in the wayfinding phase; since it is beyond the focus of the current study, we refer to the study by Karimpur and Hamburger (2016) for performance differences between congruent (presentation in the identical modality) and incongruent (presentation in a different modality) landmark presentation in human wayfinding]. The participants then had to choose between three directions (go straight, make a left turn, and make a right turn) via verbal response and/or registration by hand (to control for possible difficulties telling left from right. Regardless of whether participants answered correctly or not, the wayfinding video continued in the correct (learned) direction.

To test landmark recognition, a *recognition task* followed in which the 18 landmarks of the learning phase and additionally 18 distractors were presented in randomized order in the same modality as in the learning phase (Fig. 1). In order to control for position effects, we generated six alternating randomized stimuli presentations for the recognition task (see Online Resource 3). When the stimulus appeared, the participants had to indicate verbally whether they saw the image or smelled the odor in the previous learning phase. Participants were asked to respond as fast but correctly as possible. One month later (t2), participants were asked to do the same wayfinding and recognition task as at t1 again. The route, stimulus sequence, and stimulus modality of both tasks were the same as at t1. Therefore, participants did not repeat the learning phase at t2 again. Additionally, at the end of t2, participants answered a questionnaire including demographic questions (i.e., age, gender, level of education, and occupation), strategy usage of remembering the route, and prior experience with olfactory and navigation experiments.

Existing studies and a preliminary study conducted by us with a smaller sample have shown that visual landmarks in particular lead to a nearly 100% accurate recognition performance (Karimpur and Hamburger 2016) resulting in ceiling effects. Therefore, the design of the present study focuses more on the results of the wayfinding task since the wayfinding phases of the previous studies did not show any ceiling effects. Moreover, we aim for a greater significance of the wayfinding results than of the recognition results, as wayfinding tasks provide valuable information about spatial orientation, whereas the recognition task solely assesses landmark knowledge. However, landmark knowledge is as important as direction memory during landmark-based wayfinding and is often used as a measure of orientation performance in the previous studies (Cheng et al. 2022; Presson and Montello 1988; Siegel and White 1975; Stites et al. 2020). For this reason, we still perform a recognition task after the wayfinding task. However, we chose this order of experimental phases based on our prioritization for the wayfinding task results, as it does not allow for any confounding effects for the wayfinding phase. As a consequence, we have to accept that the recognition phase is confounded by the repeated presentation of the landmarks in the prior wayfinding task. This is especially relevant for the second time of testing since results of the recognition task will not be able to show long-term memory effects. Accordingly, the results will be interpreted with reservations in the discussion.

## Results

Our experiment used a two-factorial design with the between-subject factor modality (olfactory vs. visual) and the within-subject factor time (t1: right after learning phase vs. t2: 1 month after learning phase). In the wayfinding and the recognition task, performance was assessed as percentage of correct decisions. For this, the proportion of correct route decisions was used in the wayfinding task; in the recognition phase, the sum of correct rejections of distractors and hit rates for landmarks was determined. To allow a better comparability with existing studies in human wayfinding, the performance is calculated in percentage instead of absolute numbers, analogous to those studies (e.g., Arena and Hamburger 2022; Balaban et al. 2017; Hamburger and Knauff 2019). In the following, we only report results comparing the unimodal conditions, namely, olfactory versus visual modality. For all results, significances, as well as effect sizes, are reported. The test assumption of normal distribution tested with Kolmogorov–Smirnov tests was given for all conditions at all times. Further, Levene tests showed equal variances for all conditions. In case multiple testing, t-tests were Bonferroni corrected. Welch’s t-tests are reported. IBM SPSS Statistics Version 29.0.0.0 was used for analysis and to create all figures in the results section.

### Wayfinding

At both time points, the participants performed a wayfinding task (procedure described above).

#### Visual versus olfactory

**Fig. 2:**
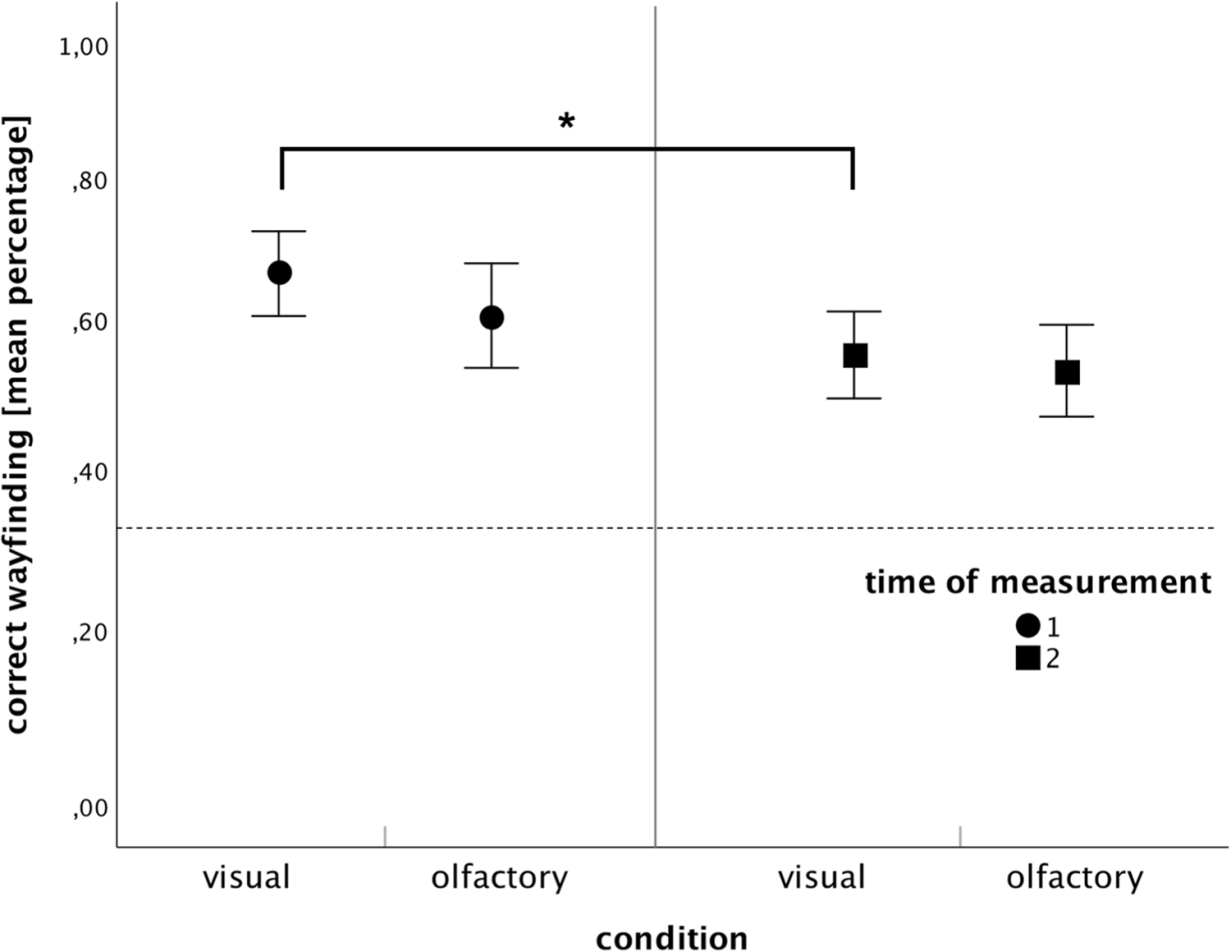
Mean wayfinding performance (correct responses, in %) for visual and olfactory stimuli for t1 and t2 (error bars = SEM) with significant differences after pairwise comparisons. *p < .05. The chance level is indicated by the dotted line

For the question whether the wayfinding performance varies between the different modalities (olfactory vs. visual) right after the learning phase (t1) and 1 month later (t2), the following results were obtained. Looking at the means, wayfinding performance for the visual condition (*M* = 0.66, SEM = 0.15 for t1; *M* = 0.55, SEM = 0.15 for t2) was higher than for the olfactory condition (*M* = 0.59, SEM = 0.16 for t1; *M* = 0.53, SEM = 0.15 for t2) at both times of testing. These findings are visualized in Fig. 2. An ANOVA with repeated measurement with the dependent variable “wayfinding performance” and the independent variable “modality” showed a significant main effect for the factor “time” [time: *F*(1,49) = 13.623, *p* < 0.001, *η* = 0.218] but no significant effect for either “modality” or the interaction “modality x time” [modality: *F*(1,49) = 1.669,* p* = 0.202, *η* = 0.033; interaction: *F*(1,49) = 1.332,* p* = 0.254, *η* = 0.026]. Further, the collected data were analyzed using independent two-tailed t-tests and a non-parametric Mann–Whitney U-test which revealed no significant differences between the olfactory and visual conditions, both right after the learning phase and 1 month later [t1: *t*(50) = 1.250, *p* = 0.217, *d* = 0.167; *U* = 263.00, *Z* = −1.374, *p* = 0.169; t2: *t*(49) = 0.120, *p* = 0.623, *d* = 0.152; *U* = 289.50, *Z* = −0.654, *p* = 0.513].

In general, it turns out that navigating with olfactory landmark information is possible as well, since performance is significantly above chance level at all testing times [> 33%; t1: *t*(24) = 7.52, *p* < 0.001, *d* = 0.18; t2: *t*(23) = 6.33, *p* < 0.001, *d* = 0.15].

#### Memory effects

In addition to comparing the olfactory and visual condition, comparisons were also made between the times of testing [immediate (t1) and 1 month later (t2)]. Wayfinding performance in both conditions was higher immediately after testing than 1 month later. Two-sided paired sample t-tests and a Wilcoxon test revealed significant differences between t1 and t2 for the visual condition [*t*(26) = 3.690, *p* = 0.001, *d* = 0.158; *z* = −3.049, *p* = 0.002] but no significant differences between t1 and t2 for the olfactory condition [*t*(23) = 1.666, *p* = 0.109, *d* = 0.173; *z* = −1.585, *p* = 0.113].

#### Sequential learning

A possible explanation for the differences in wayfinding performance between the olfactory and visual conditions could be a difference in participants’ learning strategies. Two alternative explanations are possible: spatial sequence learning (SSL; e.g., Deroost and Soetens 2006; Keele et al. 2003) and landmark-based learning. If participants used the spatial sequential learning strategy, they would have learned only the directional information without a connection to the presented landmark information. Since sequential learning is closely linked to the primacy and recency effect, participants would have used this strategy especially for the first three and the last six intersections according to Murdock (1962) and Baddeley and Hitch (1977). To test whether the use of learning strategies differed between both conditions, we conducted independent two-tailed t-tests comparing the wayfinding performance of the first three and last six responses between both conditions (visual and olfactory) over both times of measurement. Since no significant differences in wayfinding performance were found [first three: *t*(49) = 0.168, *p* = 0.867, *d* = 0.196; last six: *t*(49) = 2.003, *p* = 0.051, *d* = 0.176], we concluded that the use of SSL did not differ between both groups and can be ignored in the analyses.

### Recognition

At both time points, the participants also performed a recognition task (procedure described above).

#### Visual versus olfactory

**Fig. 3:**
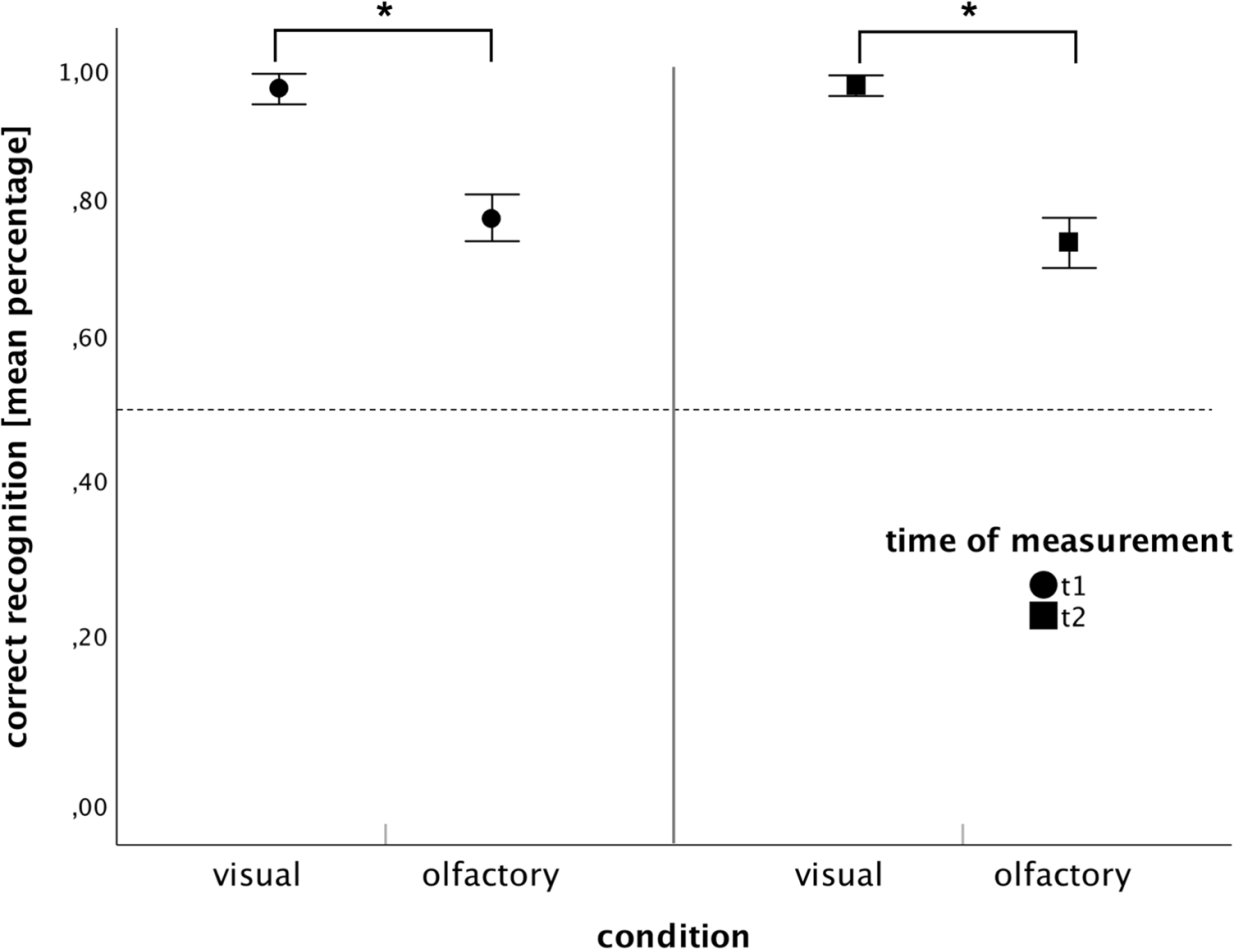
Mean recognition performance (correct responses, in %) for visual and olfactory stimuli for t1 and t2 (error bars = SEM) with significant differences after pairwise comparisons. *p < .05. The chance level is indicated by the dotted line

To test whether recognition of olfactory landmark information differs from visual landmark information right after the learning phase (t1) and 1 month later (t2), hits and correct rejections of distractors (correct responses) across the recognition phase were analyzed. Visual landmark information (*M* = 0.97, SEM = 0.063 for t1,* M* = 0.96, SEM = 0.080 for t2) was better recognized than olfactory landmark information (*M* = 0.76, SEM = 0.076 for t1, *M* = 0.75, SEM = 0.103) at both times of testing. These findings are visualized in Fig. 3. An ANOVA with repeated measurement was conducted and revealed a significant main effect for the factor “modality” [*F*(1, 48) = 115.477, *p* < 0.001, *η* = 0.706] but no significant main effect for “time” or the interaction “modality x time” [time: *F*(1,48) = 0.619, *p* = 0.435, *η* = 0.013; interaction: *F*(1,48) = 0.035, *p* = 0.852, *η* = 0.001]. This difference between the olfactory and visual conditions is significant for both times of testing as independent two-tailed t-tests and Mann–Whitney U-tests revealed [t1: *t*(50) = 9.440, *p* < 0.001, *d* = 0.076; *U* = 41.50, *Z* = −5.528, *p* < 0.001; t2: *t*(48) = 8.188, *p* < 0.001, *d* = 0.091; *U* = 45.50, *Z* = -5.237, *p* < 0.001].

In general, recognition was above chance level for both conditions at both testing times (> 50%).

#### Memory effects

Similar to the wayfinding performance, comparisons were also made between the times of testing [immediate (t1) and 1 month later (t2)]. Two-sided paired sample t-tests and Wilcoxon tests revealed no significant differences between t1 and t2 for neither the visual condition nor the olfactory condition [visual: *t*(26) = 0.463, *p* = 0.647, *d* = 0.082; *z* = −0.213, *p* = 0.831; olfactory: *t*(22) = 0.631, *p* = 0.535, *d* = 0.090; *z* = -0.973, *p* = 0.330].

## Discussion

In general, the results indicate that olfactory cues only can be used to aid navigation since participants made on average 60 percent correct route decisions at both times of testing (64% at t1 and 55% at t2) which is significantly above chance level (one-third). Thereby, wayfinding performance in the olfactory condition did not differ significantly from the visual condition at both times of testing. However, participants who used visual cues performed significantly worse 1 month after learning (t2) compared to their performance immediately after learning (t1), whereas performance in the olfactory condition did not decline over time. Looking at the recognition task, it turns out that visual landmarks were recognized better than olfactory landmarks right after learning and 1 month later.

### Odors as landmarks

First, our results suggest that humans can indeed use their sense of smell for successful navigation through their environment. Based on the present results, it cannot be said that odors are of any relevance for human wayfinding, but that they *can be* significant because in the standardized laboratory setting, humans were basically able to use olfactory cues for orientation. Appleyard (1969), Carr and Schissler (1969), and Siegel and White (1975), among others, have stated or implied the definition of “a landmark [being] any distinct object or feature that is *noticed* and remembered” (Presson and Montello 1988, p.378). According to this definition, the sense of smell would thus be virtually meaningless in human wayfinding because we are often unable to report, label, or consciously perceive odors. However, from our point of view and from the previous findings, landmarks do not necessarily need to be consciously recognized and remembered. Findings suggest that olfactory information needs to be *differentiable,* but not *identifiable,* for successful recognition (Hamburger and Knauff 2019). We believe that humans use their sense of smell rather implicitly in contrast with other senses such as the visual sense, whose information we process in a more explicit manner. This could also account for other modalities such as the human auditory system. Moreover, the implicit processing of odors could be an explanation for why initial retention is worse at t1 since implicit processing implies that information is processed with less depth. Therefore, it also leads to less interference, and as a result, the acquired information remains stable with minimal loss over time. Since there is a lack of the literature on this topic, we would like to highlight its importance and include it in the future research.

Our finding of humans being able to use olfactory cues for navigation is consistent with the results of Hamburger and Knauff (2019), who reported 64 percent correct route decisions immediately after the learning phase. One could argue that this results only from spatial sequential learning (e.g., Deroost and Soetens 2006) and not from correct recall of the landmark information as directional cue. However, the previous studies from our research group such as Hamburger and Knauff (2019), Hamburger and Röser (2014), or Balaban et al. (2014) compared a wayfinding task using olfactory landmark information with a control condition in which participants were “beamed” (i.e., teleported) to random intersections of the route, then were presented with the corresponding odor, and had to make the directional decision of this specific intersection. Performance in the wayfinding and control phase was similar, indicating that odors were encoded as landmarks and were used in a map-like mental representation to make adequate route decisions (Hamburger and Knauff 2019).

Although the present study design did not include such a control condition, the experimental setting was mostly similar to that of Hamburger and Knauff (2019). Since they found no results that could be explained by SSL, we believe that the participants in the present study most likely used cognitive maps including landmarks to find their way through the virtual environment as well. Moreover, we opted not to include a control condition without any landmarks because in such a scenario, participants would only be able to rely on memorizing the route as a sequence of verbal cues such as “left, right, left” and so forth. This cognitive approach would differ significantly from the strategy employed when landmarks are present at decision points (Balaban et al. 2014; Hamburger and Knauff 2019; Hamburger and Röser 2014; for a critical discussion see also Hamburger 2020). Thus, it would not provide us with insights into whether participants perform better with olfactory landmarks. Furthermore, in our experimental design, it is not possible to include an intraindividual comparison between landmark-based wayfinding and non-landmark-based wayfinding, since at least at t2, the subjects would have prior knowledge about the landmarks. Nevertheless, to control whether the differences found were indeed due to different modalities in landmark-based wayfinding, we tested whether SSL was statistically different between the two conditions. Sequential learning occurs at the first and last intersections of the labyrinth according to the recency and primacy effect. For this, we statistically compared the first three and last six route decisions of our experiment between the visual and olfactory conditions. We report here the results of the first three and last six route decisions analogous to existing literature on the primacy and recency effect (Murdock 1962; Baddeley and Hitch 1977), but further statistical comparisons (not reported here in detail) with varying numbers of the first and last intersections showed the same results. Since we did not find any differences, we assume that even if participants used SSL or a combination of SSL and landmark-based wayfinding, the use of these methods was unlikely to differ between the two conditions and, therefore, can be ignored in the interpretation of the present results. This statistical control analysis does not show that subjects learned exclusively in a landmark-based manner, but only that if they learned sequentially, it did not differ between groups, and thus, the group differences found were due to landmark-based learning and not SSL. However, our questionnaire data from subjects as well as anecdotal reports from the previous studies (e.g., Hamburger and Knauff 2019) showed that few subjects reported learning sequentially.

Our findings show that olfactory cues are sufficient to guide navigation in the absence of other cues. In cases of blindness or other visual impairment, olfaction may even be fundamental for orientation. However, more studies are needed to investigate whether olfactory cues are spontaneously relied upon to serve wayfinding in real-world circumstances. In addition, the issue of reliability in a natural environment needs to be further investigated, as the perception of odor marks can be influenced by several factors such as wind direction, interference with other odors, and low consistencies (Koutsoklenis and Papadopoulos 2011).

### Multimodal utilization of cognitive maps

Even though it may seem intuitively plausible that the representation of a cognitive map with landmarks would be easier to be created from visual information, we argue against the close association of visual information and propose a rather multimodal utilization of cognitive maps, given the following results of recent research and our own findings of the present study.

Karimpur and Hamburger (2016), Hamburger and Knauff (2019), and Arena and Hamburger (2022) revealed almost equal wayfinding performance for all types of landmarks, regardless of the modality in which they were presented. Participants made on average 64–73% correct route decisions using olfactory landmark information (Hamburger and Knauff 2019; Arena and Hamburger 2022), when using acoustic landmarks, their performance was on average 71–85%, compared to 66–88% correct route decisions when using visual landmarks and 87% correct responses when using written words as cues (Karimpur and Hamburger 2016; Arena and Hamburger 2022). Following these results, our study shows the same pattern, finding no significant difference in the wayfinding task between the visual and olfactory conditions at both times of testing. All of the studies listed also found no significant differences in correct route decisions between the different modalities (Karimpur and Hamburger 2016; Hamburger and Knauff 2019; Arena and Hamburger 2022). This makes a multimodal use of cognitive maps, where our senses work together rather than as separate entities, even more plausible (e.g., Karimpur and Hamburger 2016). Findings in this direction already exist, but could not yet be confirmed by initial studies in our research group (Arena and Hamburger 2023).

Nevertheless, we do not doubt that our visual system provides us with the most important and prominent access to our environment. However, we want to highlight our assumption of humans using all their available senses more or less explicitly in order to optimize their orientation in everyday life. Therefore, future studies should definitely consider a rather multimodal approach on human navigation instead of overinterpreting the experiments on unimodal visual wayfinding and still relying on a visual superiority effect in human navigation (Presson and Montello 1988).

### Odor memory as a separate memory system

One month after the leaning phase, wayfinding performance between the visual and olfactory conditions did not differ significantly from each other. That means that participants did not perform better when given olfactory landmarks compared to visual cues. However, visual landmark-based wayfinding performance significantly declined from t1 to t2 whereas wayfinding performance did not decline from t1 to t2 in the olfactory condition. As our second main finding, this suggests that memory of the route was more resistant to decline when participants used odors rather than pictures as landmark information—a result that is consistent with our assumption about odor memory being a separate memory system. In 1996, Herz and Engen already postulated odor memory as being “governed by specific and distinct rules and underlying mechanisms” (p.309).

Moreover, Herz (1998) found that odors were equivalent in their ability to elicit accurate memory recall compared to verbal, visual, tactile, and musical stimuli. However, the odor-evoked memories were always more emotional. Furthermore, a study using functional magnetic resonance imaging (fMRI) compared activated brain regions during memory recall which was triggered by olfactory versus visual cues (Herz et al. 2004). Neuronal responses demonstrated that when odor cues were used, the amygdala and hippocampal regions showed significantly greater activation than for any other cue (Herz et al. 2004). Additionally, behavioral responses confirmed this since odor-evoked memories were reported as most emotional (Herz et al. 2004). Based on her findings, Herz (1998) concluded that odors are not superior reminders compared to other sensory stimuli but evoke higher emotional saliency rather than accuracy.

Both studies indeed demonstrate higher emotional saliency for odor-evoked memories. However, in Herz’ study (1998), the time between encoding and retrieval was only 48 h, and in the fMRI study (Herz et al. 2004), retrieval occurred immediately after encoding. We believe that real differences in memory retrieval between different modalities can only be revealed using a longer time period between encoding and retrieval. Therefore, we set the period between t1 and t2 to 1 month in the present study. The use of a longer retention interval could explain our results, which in comparison with Herz’ (1998) findings in fact show differences in retrieval accuracy when using visual versus olfactory memory cues.

We believe that the higher emotional saliency of odors leads to a more resistant long-term memory of the route. Therefore, at t1, odors do not result in better wayfinding than pictures since information is (possibly) rather retrieved from working memory. However, at t2 1 month later, the emotional saliency seems quite valuable since wayfinding performance does not decline when using olfactory cues. Hence, in future studies on human wayfinding, attention should be paid on how long information is retained in memory.

The study we conducted is the first research known to the authors that was able to demonstrate the unusual resistance toward decay of odor memory (Herz and Engen 1996) in the form of behavior, i.e., in human wayfinding. Even without neuroimaging techniques, the participants in our experiment exhibited behavior consistent with theories of odor processing, such as the suggested explanation by Engen (1987) and Lawless (1978) of odors being represented unitary in memory and therefore resulting in minimal loss over time due to low rates of influence (see *1.3 Odor Memory*). In doing so, we were able to reveal an odor superiority effect in terms of long-term memory for the correct route in human wayfinding, which is likely due to the higher emotional saliency of odors compared to pictures.

### Recognition does not equal wayfinding

Lastly**,** we investigated whether visual or olfactory landmarks result in better recognition at both times of testing. Here, results showed that, when it comes to landmark recognition, visual landmarks are better recognized than olfactory cues. This result stands in contrast with our findings in the wayfinding task, where no significant performance differences were found between visual and olfactory landmark information.

One reason for these contrasting findings for the recognition and wayfinding phase could be the design of our study. In the recognition task, participants had to answer as fast and correctly as possible (see *2.3 Procedure*). This resulted in participants taking very little time to decode the presented olfactory or visual stimuli as distractors or landmarks. Studies show that response times of the olfactory system range from 600 to 1200 ms (Cain 1976) whereas participants can respond to visual stimuli in only 100 ms (Posner and Cohen 1984). The temporal reaction of pictures is, therefore, 12 times faster than for odors. Given the task design, this could have led to advantages in the correct detection of visual stimuli, as participants did not take enough time to process the olfactory stimulus and thus failed in giving correct responses.

Since the data from the wayfinding task differed across the two conditions (olfactory and visual) from the data of the recognition task, it shows that wayfinding tasks do not equal recognition tasks (i.e., Hamburger and Röser 2014 revealed similar results). Correct recognition of a stimulus alone does not predict correct wayfinding but is often used exclusively to test human navigation skills (i.e., Abu-Obeid 1998; Choi et al. 2016). The underlying cognitive processes in wayfinding and recognition are likely to be different.

However, it should be emphasized that when interpretating the data from the recognition task, special attention must be paid to the experimental design of the present study. The data collected during the recognition phase at t2 cannot be used to draw any conclusions about long-term memory, as the landmarks that are tested in the recognition phase were already partially presented in the previous wayfinding phase. In fact, the repeated presentation of the stimuli 1 month later in the wayfinding phase, just before the recognition phase, primarily relies on short-term memory retrieval in the recognition task. As a result, the present design does not allow for any conclusions regarding long-term memory with respect to the recognition of landmarks. Moreover, it is not possible to draw comparisons between the decline in wayfinding performance in, for example, in the visual condition from t1 to t2, and a potential decline in recognition performance.

However, we conclude that recognition and wayfinding must be interpreted separately and cannot be reconciled with wayfinding. Odor memory appears to coexist with memory for other senses, but our task design was only able to demonstrate a long-term memory odor superiority effect in our wayfinding task in terms of unusual resistance. Moreover, the existing literature suggests that odors are likely to be processed implicitly rather than explicitly, as the latter is the case with other senses such as vision (Degel and Köster 1999). This needs to be further investigated in the future studies.

### Limitations and implications for future research

In the following, we will discuss some methodological limitations of the present study and provide implications for future research.

The first potential methodological problem is our chosen retention interval of 1 month between t1 and t2 in order to detect long-term odor memory effects. The previous studies on long-term odor memory revealing retention functions with essentially zero slope used time intervals of 1 month or 1 year (Engen and Ross 1973; Jones et al. 1978; Lawless and Cain 1975). On the other hand, Murphy et al. (1991) found no significant difference in long-term memory performance between odors and symbols/faces after 6 months. Perkins and Cook (1990) even reported a significant decline in long-term odor memory after 1 week. As these contradicting findings give little guidance in choosing the optimal retention interval, we aimed to make the period as long as possible in order to make sure to allow for sufficient memory loss. However, due to the time and cost constraints, it was not possible to extend the time interval any further. Our results show that our chosen retention interval was indeed long enough to reveal differences between long-term odor and pictorial memory. However, future research should extend and vary time intervals between encoding and retrieval to investigate those findings more precisely.

Moreover, our results rely on t-tests for dependent samples only. However, the interaction of the ANOVA with repeated measures was not significant. The results of the study must, therefore, be interpreted with caution, and the significance of the results remains limited for now. Therefore, additional studies with more participants are needed in the future in order to support or falsify the present results.

Since the experimental design requires the olfactory cues to be presented by hand by the experimenters, the distance to the nose and the diffusion of the odors in the air are not 100 percent uniform. Therefore, the intensity of the odors may vary from subject to subject, which may affect reliability of the experiment. For more precise results, future research could use an olfactometer (Lorig et al. 1999).

Furthermore, in the wayfinding task, participants did not experience any negative effects of being lost. Consequently, it remains uncertain whether participants would be equally capable to get back on the route by relying on olfactory or visual landmarks. Future research could employ an interactive design for the wayfinding task to explore this question in detail.

Given our results, humans are likely to use all their available senses to orientate themselves. Future studies, therefore, should investigate how olfaction interacts with other senses in human wayfinding. For that, visual, acoustic, haptic, and olfactory stimuli should be used to explore how they complement or compete with each other.

Moreover, a theoretical model is needed to integrate the numerous puzzle pieces of the existing literature—with its (somewhat) ambiguous findings, common theories, and relevant approaches to navigation and olfactory research into a broader and more accurate picture of sensory processing and the use of multimodal landmarks in human spatial orientation.

Furthermore, due to technological progress, virtual environments are increasingly used in everyday life, e.g., in video games (Karimpur and Hamburger 2015), but also in research, such as in the present study. Especially in research, increasing use of virtual environments raises the question of ecological validity (Hamburger and Knauff 2019). Lloyd et al. (2009) already investigated the equivalence of route-learning performance between real and virtual environments. Their results revealed comparable error rates for wayfinding performance and no differences in strategy usage, indicating that even simple virtual desktop environments provide a useful tool for evaluating and researching navigation. With the presentation of our 3D maze on VR glasses, we increased ecological validity compared to a simple desktop presentation by providing an egocentric, first-person perspective to the participants. Even though, the existing literature supports the methodological approach used in the present study, further studies should transfer our experiment into reality with participants experiencing the visual and olfactory stimuli in an actual real environment. This way, participants are likely to focus more on the olfactory cues, as the presentation of our virtual maze on VR glasses might have caused them to concentrate more on visual information than they would in real life.

Finally, it is important to emphasize that this study is intended to be basic research, not applied research. The goal is to explore the underlying processes of our sensory modalities in wayfinding, not how these modalities are used in everyday navigation. Therefore, it is not necessary for this question to create a virtual environment “as close to reality as possible,” where one could, for example, take a wrong turn and then have to find the right way back. To answer the question whether olfactory stimuli can in principle help human orientation over a longer period of time, our laboratory setting is sufficient. In order to answer application research related questions, a more complex experimental setting with higher external validity would have to be used.

## Conclusion

With this study, we once again demonstrate that humans are able to use olfactory cues as landmark information in wayfinding. We, therefore, want to highlight the necessity of considering different modalities when studying human spatial orientation. In our opinion, only when considering more than just the visual modality, research will be able to achieve a more comprehensive understanding of the underlying cognitive processes in human spatial orientation. Further, we could show that wayfinding performance using olfactory cues is more resistant to memory decline than when using visual cues. This is a novel result that adds a new aspect to the literature on odor memory and human wayfinding. Moreover, this study supports the assumption that odor memory is a separate system (see also Herz and Engen 1996) and offers new findings on the theory of odor superiority effects in human memory. Future studies could additionally take fMRI or neural analyses into account to provide further information about the underlying cognitive processes of visual and olfactory landmark-based wayfinding and its memory effects. Finally, our findings could potentially help to explain differences in performance between landmark recognition and wayfinding. Converse results in the wayfinding and recognition tasks indicate that these tasks should be interpretated separately and are processed in different cognitive systems (see also Balaban et al. 2017). However, due to methodological issues, this finding should be interpretated with caution.

For future application purposes, understanding landmark-based wayfinding offers tremendous potential such as route navigation systems or signage (e.g., Balaban et al. 2017). Therefore, researchers should consider our suggestions in future studies to further investigate the role of different modalities and methodological approaches in landmark-based human wayfinding.

### Supplementary Information

Below is the link to the electronic supplementary material.

Supplementary file1 (PDF 247 KB)

## Data Availability

The material of the experiment is available in the supplementary material. Additional material and data will be provided by the authors on request.
