# Supplementary material for: Memory effects of visual and olfactory landmark information in human wayfinding
Source: Cogn Process. 2023 Nov 30;25(1):37–51. doi: 10.1007/s10339-023-01169-7 (PMC10827900; doi:10.1007/s10339-023-01169-7)
Supplement: Supplementary file 1 — Supplementary file1 (PDF 247 KB) [file 10339_2023_1169_MOESM1_ESM.pdf]

Supplemental material for the Journal of Cognitive Processing

Memory effects of visual and olfactory landmark information

in human wayfinding

1. Visual and Olfactory Landmarks and Distractors and Corresponding Example Pictures

The visual landmarks were taken from private sources and the license free stock images provider pexels.com.

Landmarks: Fish, Salami pizza, Nail polish, Alcohol, Pineapple, Gras, Aniseed, Fresh Laundry, Eucalyptus Pepper, Curry, Citron, Vanilla, Aftershave, Tangerine, Strawberry, Basil, Cocoa

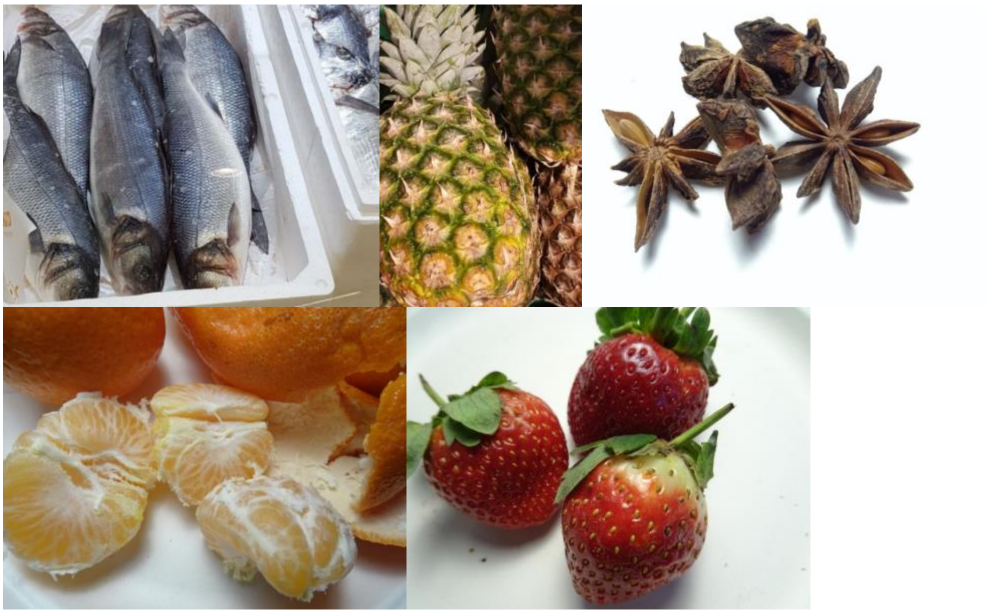

Distractors: Garlic, Vinegar, Leather, Spruce needle, Peanut, Frankincense, Coke, Lavender, Melon, Clove, Peppermint, Coconut, Banana, Apple, Rose, Cinnamon, Licorice, Black Tea

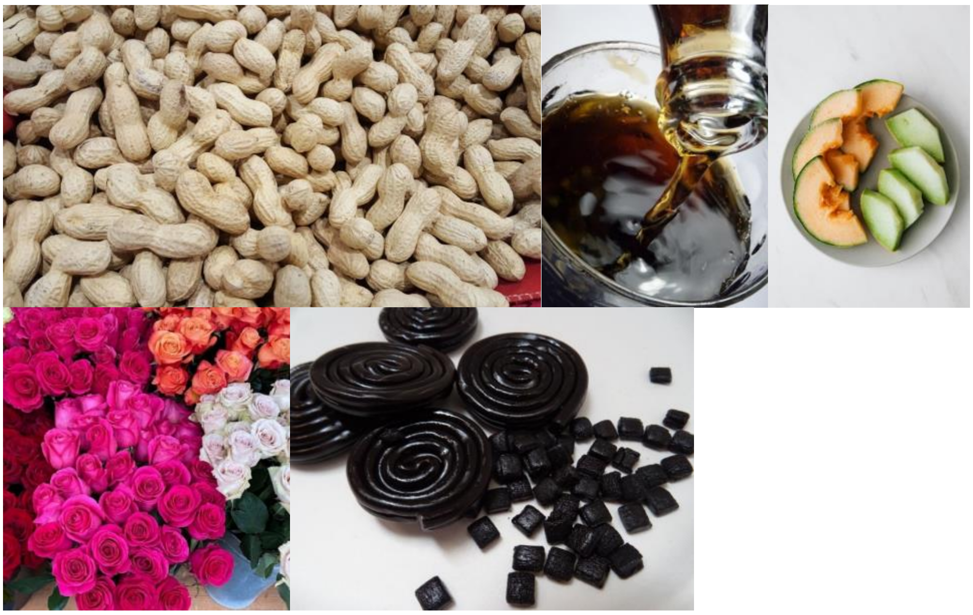

2. Stimuli Sequences for each of the two Routes

Sequence 1: Fish**,** Nail polish, Pineapple, Salami Pizza, Aftershave, Alcohol, Anisseed, Cinnamon, Curry, Fresh Laundry, Pepper, Citron, Tangerine, Strawberry, Eucalyptus, Vanilla, Basil, Cocoa

Sequence 2: Vanilla, Tangerine, Basil, Eucalyptus, Nail polish, Strawberry, Salami Pizza, Cinnamon, Fish, Aftershave, Pineapple, Anisseed, Alcohol, Citron, Cocoa, Curry, Pepper, Fresh Laundry

Sequence 3: Pepper, Cocoa, Fresh Laundry, Curry, Basil, Citron, Eucalyptus, Strawberry, Tangerine, Vanilla, Salami Pizza, Anisseed, Alcohol, Pineapple, Aftershave, Nail polish, Cinnamon, Fish

3. Recognition Task Presentations (Distractors = italics)

Recognition 1: *Clove*, *Leather*, Fish, Tangerine, Curry, *Coconut*, *Garlic*, Salami Pizza, *Spruce needle*, Cocoa, *Peppermint*, Nail polish, *Peanut*, *Licorice*, Gras, Strawberry, Eucalyptus, *Lavender*, Citron, Pineapple, Aftershave, *Coke*, Pepper, *Banana*, *Vinegar*, Vanilla, Basil, Alcohol, *Frankincense*, *Melon*, Fresh Laundry, Aniseed, *Apple*, *Rose*, *Cinnamon*, *Black Tea*

Recognition 2: *Licorice*, Vanilla, *Rose*, Tangerine, Citron, *Vinegar*, Nail polish, *Leather*, *Garlic*, Fresh Laundry, Eucalyptus, *Spruce needle*, Cocoa, *Black Tea*, Gras, *Peppermint*, Pepper, *Melon*, Strawberry, *Apple*, Basil, *Coconut*, Aniseed, *Lavender*, *Frankincense*, Fish, Alcohol, Curry, *Cinnamon*, *Banana*, Aftershave, *Coke*, *Clove*, Pineapple, *Peanut*, Salami Pizza

Recognition 3: *Black Tea*, Vanilla, *Licorice*, *Melon*, *Garlic*, *Apple*, Salami Pizza, Pepper, Citron, *Frankincense*, Curry, Basil, *Leather*, *Banana*, *Peppermint*, *Vinegar*, *Clove*, *Lavender*, Pineapple, Cocoa, Fish, *Coconut*, *Cinnamon*, Alcohol, Gras, Eucalyptus, Nail polish, *Peanut*, Aniseed, *Rose*, Strawberry, Aftershave, *Spruce needle*, *Coke*, Fresh Laundry, Tangerine

Recognition 4: Fresh Laundry, *Lavender*, Cocoa, Basil, Nail polish, *Spruce needle*, Pepper, *Peppermint*, Vanilla, *Coconut*, *Garlic*, Citron, *Black Tea*, Eucalyptus, Aftershave, *Rose*, *Banana*, Aniseed, Gras, *Apple*, *Vinegar*, Pineapple, Fish, *Melon*, *Clove*, Alcohol, Salami Pizza, Curry, *Frankincense*, Tangerine, *Peanut*, *Cinnamon*, *Leather*, Strawberry, *Licorice*, *Coke*

Recognition 5: Pineapple, *Rose*, *Garlic*, *Banana*, Aftershave, Nail polish, *Clove*, *Coconut*, *Spruce needle*, *Black Tea*, *Vinegar*, Curry, *Melon*, Fish, *Peppermint*, Gras, Strawberry, Pepper, *Apple*, Cocoa, *Coke*, *Licorice*, Salami Pizza, Basil, *Lavender*, Fresh Laundry, *Frankincense*, Vanilla, Aniseed, Tangerine, Citron, *Peanut*, *Leather*, *Cinnamon*, Alcohol, Eucalyptus

Recognition 6: *Rose*, Curry, *Frankincense*, Alcohol, *Clove*, Strawberry, Pepper, *Vinegar*, *Melon*, *Cinnamon*, Basil, Vanilla, *Banana*, Tangerine, Fresh Laundry, Aniseed, *Apple*, *Peppermint*, *Garlic*, *Coconut*, Eucalyptus, Fish, Aftershave, *Black Tea*, Nail polish, *Spruce needle*, Cocoa, Citron, Salami Pizza, *Coke*, *Peanut*, *Licorice*, *Leather*, *Lavender*, Pineapple, Gras
